# Fluctuating environmental light limits number of surfaces visually recognizable by colour

**DOI:** 10.1038/s41598-020-80591-9

**Published:** 2021-01-22

**Authors:** David H. Foster

**Affiliations:** Department of Electrical and Electronic Engineering, University of Manchester, Manchester, M13 9PL UK

**Keywords:** Computational biology and bioinformatics, Electrical and electronic engineering, Sensory processing, Neuroscience, Colour vision, Object vision, Pattern vision, Atmospheric dynamics

## Abstract

Small changes in daylight in the environment can produce large changes in reflected light, even over short intervals of time. Do these changes limit the visual recognition of surfaces by their colour? To address this question, information-theoretic methods were used to estimate computationally the maximum number of surfaces in a sample that can be identified as the same after an interval. Scene data were taken from successive hyperspectral radiance images. With no illumination change, the average number of surfaces distinguishable by colour was of the order of 10,000. But with an illumination change, the average number still identifiable declined rapidly with change duration. In one condition, the number after two minutes was around 600, after 10 min around 200, and after an hour around 70. These limits on identification are much lower than with spectral changes in daylight. No recoding of the colour signal is likely to recover surface identity lost in this uncertain environment.

## Introduction

Changes in natural light affect how the world appears and how we interact with it. The movement of shadows alters the perception of contour and depth^1,2^ and the setting sun makes directly lit surfaces appear more red and shadowed areas more blue^3,4^. These spatial and spectral variations are evident over intervals of an hour or more. But there are other changes in illumination that have a shorter time scale and which might also affect appearance.

Even in a visibly cloudless sky, the solar beam undergoes randomly varying attenuation from unobservable cirrus clouds and aerosols, including sulphate, black carbon, organic materials, and dust^1,5,6^. The intensity variations over intervals of minutes are small, of the order of 0.1% around midday, well below the threshold for visual detection^7^, though they do increase around dawn and dusk^8,9^. Yet there is an important difference between these fluctuations in the light incident on a scene and the fluctuations in the light reflected by its constituents, especially the three-dimensional, spatially and spectrally complex surfaces of natural scenes^10–12^. Are the changes in reflection large enough to impair the visual recognition of surfaces by their colour, and if they do, by how much?

A straightforward way to address these questions is to measure, directly or indirectly, the perceived or apparent colour of surfaces in a scene before and after an illumination change^13,14^. If the difference in appearance of a particular surface is not too large, that is, within some tolerance defined by the observer’s sensitivity or interpretation of colour differences or colour categorization^15,16^, then a correct surface match should be possible. But this approach neglects the other surfaces in the scene whose colour differences may also be within tolerance. Any one of these surfaces could offer an incorrect surface match^17,18^.

Instead, a more comprehensive approach is needed, one that accommodates the similarities of appearance in any sample of surfaces drawn from a scene and the changes of the appearance in the sample over time. Each is intrinsically uncertain.

The effect of similarity on performance is governed not just by the gamut of surface colours in a scene^19^ but also by the differing abundances or relative frequencies of different colours. These relative frequencies influence the composition of any random sample from the scene and therefore their distinguishability^20,21^.

The effect of illumination changes is usually considered in relation to the phenomenon of colour constancy, the invariance of perceived or apparent colour under changes in the intensity and spectrum of the illumination^15,16,22,23^ or with atmospheric scatter^24^. These changes in illumination are conventionally assumed to be uniform across a scene or have a smooth spatial gradient, and they can be largely offset by scaling cone signals^7,25^ or establishing correspondences between cone signals^18^. Yet real-world changes in light are more likely to be geometric—a redistribution of light over the scene—than spectral. These changes are typified by the shifting dappled illumination in forests and woodland^26,27^ under the changing path of the solar beam^1^.

Happily, there is a simple measure that is sensitive to both similarities of surface appearance and changes in appearance over time. It is the number of surfaces in a sample that can be recognized by their colour despite the illumination change; that is, more precisely, the number that can be identified as the same over the interval containing the change^17,21^. Estimates of this number can be made computationally by information-theoretic methods^28^, which provide a theoretically best possible limit, a least upper bound on the average number possible for the given conditions^18,29^. In these calculations, only the spectral properties of the light reflected from each surface in a sample are considered, not the local spatial features, for example, texture, shape, location, and proximity to other surfaces, which, unlike the illumination, are assumed to remain constant.

Source data for these estimates were taken from successive hyperspectral radiance images of 18 natural, outdoor scenes that included both undeveloped and developed stationary land cover^30,31^. Pairs of images were available separated by intervals of about one to fifteen minutes, and, for four of the scenes, multiple pairs were also available with intervals ranging up to several hours. It is stressed that the illumination changes were those actually recorded. Simulations of spectral changes in daylight were used solely as a control^21,29^.

The main outcome of this work is that even over short time intervals, fluctuations in environmental light severely limit surface recognition by colour, and much more so than with spectral changes in illumination.

## Results

### Amplifying variation by reflection

**Figure 1:**
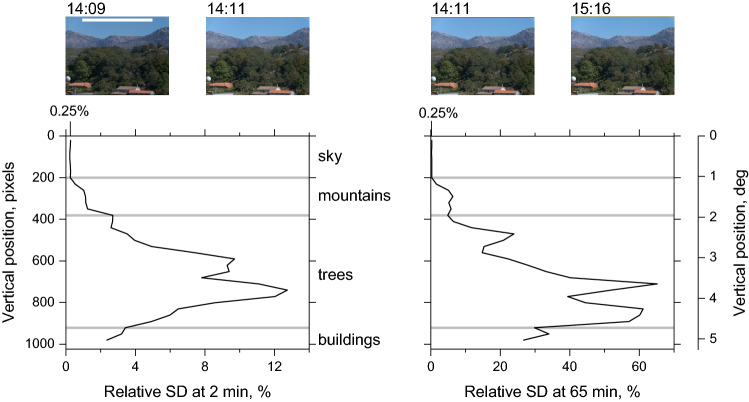
Variation in cone excitations across two different time intervals as a function of the vertical sample position. The sample area is a thin horizontal strip (leftmost image, white rectangle near top). Its vertical position is plotted on the leftmost axis in pixels and on the rightmost in degrees of visual angle. Variation is quantified by the standard deviation (SD) of the differences in cone excitations relative to the mean of the sample, plotted on the horizontal axis in per cent. Data are for a 2-min interval in the left plot and for a 65-min interval in the right plot, which has a larger horizontal scale.

As a preliminary, to illustrate the physical differences in the variation of direct and reflected light, radiance data were sampled selectively from a scene containing both sky and a mixture of landcover types. The hyperspectral radiance images of the scene were acquired at 14:09, 14:11, and 15:16 in the course of a previous study^32^. The images in Fig. 1 show colour renderings of the radiance data. Sample areas were defined by a thin horizontal strip, size 896 × 30 pixels (leftmost image, white rectangle near top). Radiance values were transformed into long-, medium-, and short-wavelength-sensitive cone excitations. The standard deviation (SD) of the differences in excitations at 14:09 and 14:11 and separately at 14:11 and 15:16 were obtained as a function of the vertical position of the strip in the 1344 × 1024-pixel image. The size of the strip was a compromise between making reliable estimates of the SD and avoiding bias.

The plots in Fig. 1 show the SD of the magnitude of the differences in cone excitations relative to the mean over the sample at each vertical sample position, plotted on the left and right axes. Data from regions of the scene with mainly sky, mountains, trees, or buildings are demarcated by horizontal grey lines. The left plot is for differences between cone excitations at 14:09 and 14:11. For direct light from the sky, the relative SD varies between 0.22% and 0.27%. These values for a 2-min interval are of the same order as those from independent pyrheliometer recordings from other scenes. For light reflected from distant mountains, the relative SD increases to about 1.1%, but from the nearer trees and buildings it is between 2.7% and 12.7%, which should be detectable by a normal trichromatic observer with sensitivity characterized by the Weber-Fechner fraction^7^ or rather larger values^33^ or by colorimetric measures^34,35^.

The right plot is for differences between cone excitations at 14:11 and 15:16. For direct light from the sky, the relative SD increases little across this larger, 65-min interval, as expected, whereas for light from trees and buildings it is an order of magnitude larger (the horizontal scale is five-times larger in this plot).

Illumination fluctuations are to be expected with plant canopies^27,36^, which add to the spatial and spectral variance^12^. Even so, foliage movement seems to contribute little to the variance in Fig. 1 since the multiplicative increase in relative SD between the left and right plots is about the same for mountains and trees. In the remainder of this analysis, samples were not limited to strips and were drawn freely from the whole image.

Representations of radiances by cone excitations are useful for quantifying the physical effects of illumination changes, but they are poorly suited to quantifying observer responses^7,25^. Equally different cone excitations do not generally imply equally distinguishable radiances.

### Numbers of distinguishable surfaces

A colour space standardized by the Commission Internationale de l'Eclairage (CIE) was used to represent radiances in a perceptually relevant way^37^. This space CIECAM02^38^ has axes corresponding to lightness, redness-greenness, and yellowness-blueness and is approximately uniform in the sense that equal Euclidean distances correspond to approximately equal perceived colour differences^39,40^. As a control, another colour space S-CIELAB^41^ was also tested, which though less uniform than CIECAM02 space, simulates the spatial-frequency filtering of the whole image by the eye^42^ and the resulting spectral mixing^43^.

**Figure 2:**
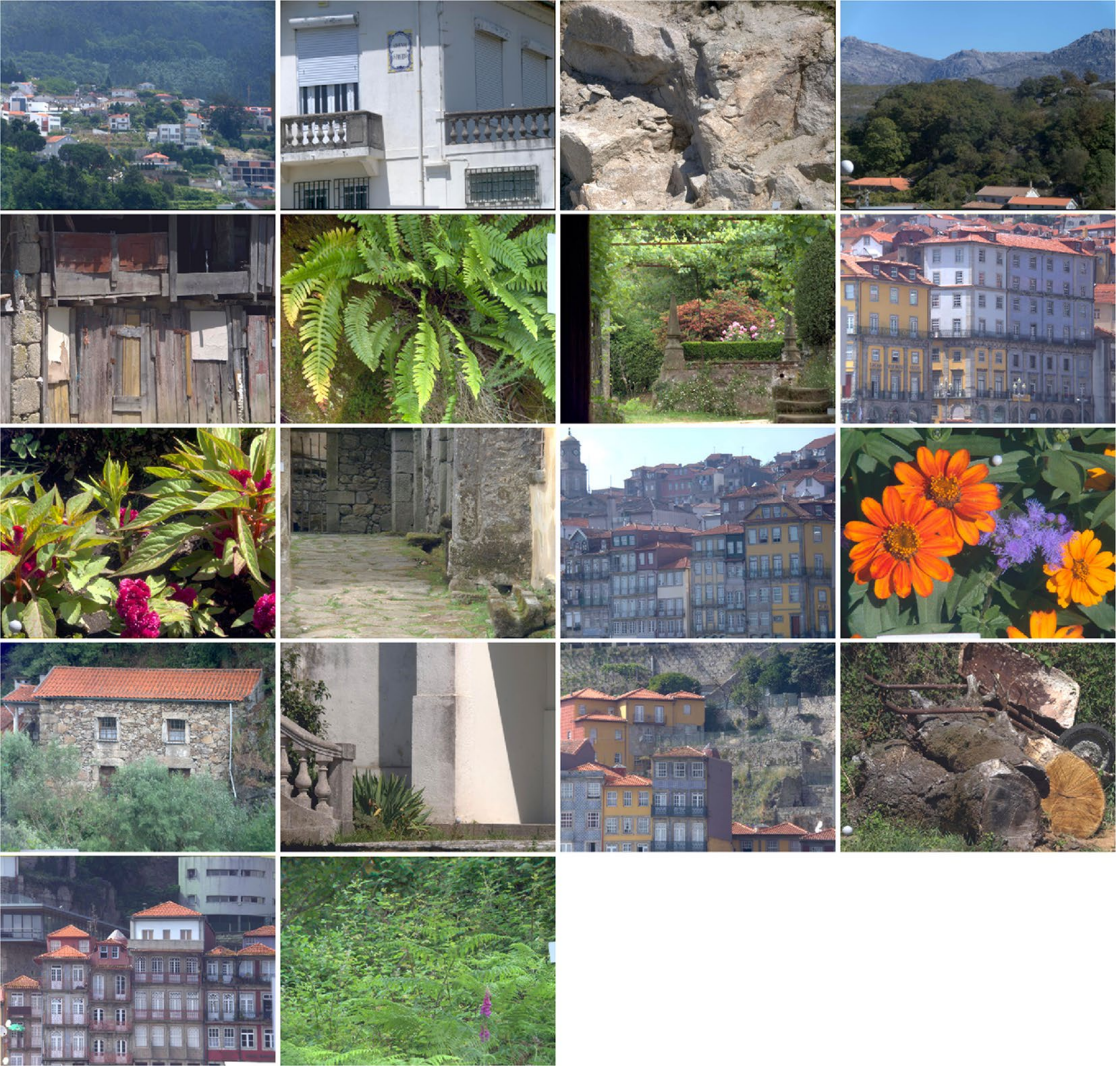
Colour images of the 18 scenes used in the analysis. Each image is from one of a pair of hyperspectral radiance images separated by a time interval of between about 1 min and 15 min (only one member of each pair is shown). For the four scenes in the top row, multiple hyperspectral image pairs were available with time intervals ranging from about 1 min to at least 4.6 h. The hyperspectral images had dimensions 1344 × 1024 pixels and spectral range 400–720 nm sampled at 10-nm intervals^12,32^.

To provide a reference level for similarities in surface appearance, estimates of the number of surfaces distinguishable by colour were obtained for single radiance images from each of the 18 scenes in Fig. 2. Although population estimates of these numbers have been made previously^20^, sample estimates are needed for the scenes used in this analysis. Estimates depend both on scene composition and on illumination. They also depend on an observer threshold, without which the number of surfaces may not be well defined^43^.

The first data row in Table 1 shows the estimated number of distinguishable surfaces averaged over the 18 scenes, with confidence limits in Supplementary Table S1 online. Values are for two models of observer internal noise, one a Gaussian distribution and the other a uniform distribution. The width of each distribution was referred to a hard discrimination threshold Δ*E*^thr^ of 0.5, equivalent to a just perceptible colour difference in CIECAM02 space^44^.

**Table 1:** Numbers of distinguishable surfaces and surfaces identifiable over time intervals.

Surface measure	Images/scene^b^	Interval	Observer internal noise^a^	
			Gaussian	Uniform
**18 scenes**^**c**^				
Distinguishability	1	–	12,000	19,000
Identifiability over intervals	2	1–15 min	270	270
**4 scenes**^**d**^				
Distinguishability	> 17	–	9500	15,000
Identifiability over intervals	> 110	2 min	580	580
		10 min	210	210
		1 h	69	69

Entries are logarithmic inverses of mutual information estimates averaged over images, image pairs, and regression estimates. Estimated 95% confidence limits are in Supplementary Table S1 online. Entries to 2 significant figures.

^a^Gaussian and uniform models of observer internal noise were referred to a hard discrimination threshold Δ*E*^thr^ = 0.5

^b^Number of images from each scene acquired at different times.

^c^All scenes in Fig. 2

^d^Top row scenes in Fig. 2.

With Gaussian internal noise, the average estimated number of distinguishable surfaces is about 12,000 and with uniform internal noise about 19,000. Both values are compatible with earlier estimates^20^. Notice that these estimates refer to the underlying radiance image, not the discrete hyperspectral sample that approximates it^21^.

With S-CIELAB space, the corresponding estimates are much smaller, about 760 and 1200, that is, about 6% of those with CIECAM02 space, for both models of observer internal noise (Supplementary Table S2 online). The reduction seems less to do with spatial-frequency filtering and more to do with the properties of CIELAB space itself, which gives much smaller estimates than CIECAM02 space (not shown here).

Doubling Δ*E*^thr^ reduced the average estimated number of distinguishable surfaces to about 1600 and 2600 with CIECAM02 space and the two models of observer internal noise (Supplementary Table S3).

Recall that these estimates take no account of illumination changes.

### Numbers of surfaces identifiable over short time intervals

What, then, are the effects of an illumination change? Estimates of the number of surfaces identifiable by their colour over intervals of about 1–15 min were obtained for single pairs of radiance images from each of the scenes in Fig. 2. Over these short intervals, the spectrum of the solar beam did not measurably change according to recordings from a neutral reference surface in the field of view^21^. The second data row in Table 1 shows the estimates.

With CIECAM02 space, the average estimated number of identifiable surfaces per scene is about 270 with both models of observer internal noise and a reference discrimination threshold Δ*E*^thr^ of 0.5. This average estimate is almost two orders of magnitude smaller than that for the number of distinguishable surfaces. Doubling Δ*E*^thr^ reduced the average estimate from about 270 to about 180 (Supplementary Table S3).

With S-CIELAB space, the corresponding average estimates differ from those with CIECAM02 space by about a factor of two (Supplementary Tables S2).

There was no reliable correlation between the number identifiable and the length of the time interval with this range of intervals.

### Long time intervals

How does increasing the interval between images beyond 15 min affect the number of surfaces identifiable by their colour? Estimates of this number for intervals $\Delta t$ ranging from about 1 min to at least 4.6 h were obtained from the four scenes in the top row of Fig. 2. For each scene, at least 100 pairs of images with different intervals were available. Colour images of the main sequences are shown elsewhere^32^. This analysis used the same methods as in the preceding section. Again to provide a reference level, estimates of the number of distinguishable surfaces were also obtained.

The third data row of Table 1 shows the estimated number of distinguishable surfaces averaged over the multiple images from each of the four scenes, with confidence limits in Supplementary Table S1 online. The average estimates of about 9500 and 15,000 for the two models of observer noise are somewhat smaller than with the set of 18 scenes in the first data row, but they come from images recorded later in the day.

**Figure 3:**
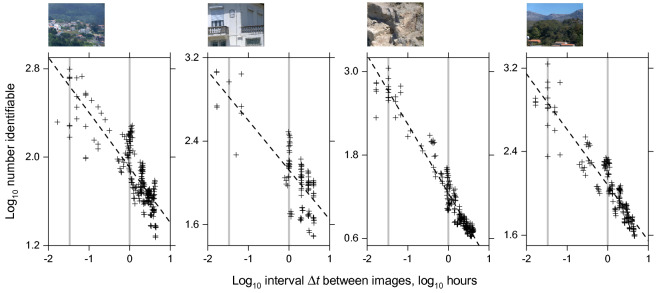
Estimated number of identifiable surfaces as a function of time interval Δ*t* between successive radiance images of each of the four scenes shown above the plots. Images from the scenes were represented in CIECAM02 colour space with a Gaussian model of observer internal noise referred to a hard discrimination threshold Δ*E*^thr^ of 0.5. Logarithmic scales are used to stabilize variance and linearize dependencies. Vertical axes are adjusted for the range of data available. The dashed lines are linear regressions and the grey vertical lines mark intervals of 2 min and 1 h.

Figure 3 shows the logarithm of the estimated number of identifiable surfaces plotted against the logarithm of the interval $\Delta t$ between images from each scene represented in CIECAM02 colour space with Gaussian internal noise and a reference discrimination threshold Δ*E*^thr^ of 0.5. The sample values of log Δ*t* are distributed nonuniformly because of the linear timing regime used in the original image acquisitions^32^. The dashed lines are linear regressions. Similar plots were obtained with the assumption of uniform internal noise and with images represented in S-CIELAB space.

If the magnitude of the illumination changes were constant with increasing interval, then the linear regressions would be flat, whereas the logarithm of the estimated number identifiable declines rapidly with log Δ*t*. The regressions account for between 63% and 94% of the variance over the approximately 4.6 h range. Still, as an explanatory variable, Δ*t* is only a proxy measure for the unspecified change in the spectral and geometric properties of the illumination. The actual change and the resulting variance about the regression line depend on the reflecting surfaces in the scene, the level of illumination, the time of day, and the differential effects of changing solar altitude and azimuth on the distribution of shadows.

Nonetheless, the regression fits can be used to estimate the number identifiable for representative values of Δ*t*, say 2 min, 10 min, and 1 h. The bottom three data rows in Table 1 summarize these estimates averaged over the four scenes, with confidence limits in Supplementary Table S1 online. With CIECAM02 space and Gaussian internal noise, the average estimated number identifiable at 2 min is about 580, falling to about 210 at 10 min, and 69 at 1 h. The same values were obtained with uniform internal noise. The value of 210 at 10 min is compatible with the average of 270 for the 18 pairs of images with intervals of 1–15 min (see confidence limits, Supplementary Table S1 online). With S-CIELAB space, average estimates varied from being smaller than with CIECAM02 space at short intervals to being larger at long intervals, due presumably to spatial-frequency filtering.

As noted earlier, these illumination changes were mainly geometric, with little or no change in spectrum.

### Spectral changes in illumination

Are the effects of changes in illumination of the kind considered here similar to those with purely spectral changes in illumination on a scene? Spectral changes are usually simulated^18,45^ since they are difficult to record naturally. To answer this question, estimates of the number of surfaces identifiable by colour were obtained under a change in a global illuminant that was equivalent to a shift in daylight spectrum from a correlated colour temperature of 6500 K, corresponding to typical daylight, to one of 4000 K, corresponding to the setting sun.

With CIECAM02 space and Gaussian internal noise with a reference discrimination threshold Δ*E*^thr^ of 0.5, the estimated number of surfaces identifiable under this spectral illuminant change was about 4400 per scene averaged over the four scenes in the top row of Fig. 2. With uniform internal noise instead of Gaussian noise, it was about 5500 per scene. Both values are manifestly greater than the corresponding values of 580 obtained with real-world changes in illumination over 2 min (Table 1).

## Discussion

The reflecting properties of materials in a natural environment vary randomly from point to point owing to variations in their composition, texture, orientation, weathering, and other factors^10,21^. The light within this environment also varies randomly from point to point, with mutual reflection, occlusion, and transilluminance producing chromatic variation extending beyond the daylight locus^12,46^. The effect of this diversity on vision is, however, moderated by the differing abundances of surface colours^20,47^. Thus the average number of surfaces in a scene that can be distinguished by their colour is of the order of 10,000, much less than the number of colours that can be distinguished within the scene^20,47^.

Even then, the number of distinguishable surfaces does not represent the number that retain their visual identity over time. Illumination and surface reflection together determine the image presented to the eye. And because natural illumination varies, even over intervals as short as a few minutes, there can be large physical changes in reflected light from some or all of a scene. The number of surfaces that can therefore be identified by their colour after an interval is much less than the number that can be distinguished. In one condition of this study, the average number that can be identified is around 600 after two minutes, equivalent to about 5% of the number distinguishable, and it falls to around 200 after 10 min, and to around 70 after an hour. Crucially, though, it is not these particular values that are significant, but the order of magnitude of the effects they represent.

These estimates may be the best possible for a normal trichromatic observer, yet they do depend on the observer model. Most obviously, increasing observer internal noise decreases both the number of distinguishable surfaces and the number identifiable after an interval. Similarly, including spatial-frequency filtering of the image by the eye with S-CIELAB colour space reduces the number of distinguishable surfaces but also reduces the rate at which the number of identifiable surfaces declines with the length of the interval. These reductions are attributable partly to the use of CIELAB colour space instead of the more uniform CIECAM02 colour space and partly to the reduction in uncorrelated variance between images.

The present findings on real-world illumination changes appear to confirm an earlier speculation^20^ that colour constancy, or the lack of it, does not generally determine the extent to which surfaces can be identified by their colour in natural scenes under different illuminants. To be clear, this speculation was based on other phenomena: the effect of relative frequency of different colours on the distinguishability of surface colours and the effect of simulated spectral changes in daylight. In fact, as shown here, real-world illumination changes have an even greater impact than relative frequency.

Still, it might reasonably be argued that the contribution of higher-level cognitive mechanisms has not been considered. There is a long history of the study of observers’ ability to separate the appearance of surfaces from judgements about them^45,48–51^, specifically, to be aware of a difference in illumination and, at the same time, of the stability of surface reflectance. Yet it is one thing to be aware of this stability and another to correctly identify individual surfaces by their colour. These two competencies are distinct, and the one does not necessarily imply the other.

There are several qualifications to this analysis. First, the information-theoretic estimates, despite providing a least upper bound on the number identifiable, do not actually indicate which surfaces are identifiable. To find those surfaces, a specific mapping from one image to the other needs to be defined, and its performance is usually imperfect^29^. Illustrations of some errors in identification with mappings defined by standard chromatic adaptation transformations are given elsewhere^21,29^.

Second, observers were assumed to behave optimally. Procedural factors were not taken into account, for example, how search for a particular surface might be implemented^52^ or affected by peripheral colour awareness^53^, attention^54^, or memory^55,56^. Observers’ search strategies can turn out to be far from optimal, with local scene colour^57^, context^58^, and salience^59^ all influencing performance. Global image properties can also affect appearance judgements^13,60,61^. As a consequence, observers presented with a possible match may accept colour differences that exceed conventional threshold values^16,18,62^. In short, the numerical limits reported here are likely to be overestimates.

Third, these limits are contingent on the chosen sample of 18 scenes. They included near, middle, and distance views drawn from the main land-cover classes^30,31^, containing shrubs, ferns, flowers, rock, stone, urban buildings, and farm outbuildings. Larger data sets might reveal different limits, though the control measurements with simulated spectral changes in daylight on the 18 scenes were consistent with those previously reported with 50 scenes^20^.

Fourth, and last, the limits are also contingent on the characteristics of the illumination variations. In the presence of cloud, changes in solar altitude and azimuth may produce changes in the pattern of reflected light qualitatively different from those considered in this analysis.

Throughout this analysis, the concern has been only with the spectral properties of the light reflected from individual surfaces in samples drawn from a scene. If, instead, observers had access to more than just spectral properties, for example, local spatial features such as texture^63^ and shape^64^, a more robust response might be achieved. But spatial features remain defined by the light they reflect, which, in turn, depends on the fluctuating incident beam. They are therefore subject to the same information-theoretic limits that affect recognition by colour. By the nature of these limits, any recoding of the colour signal, including the usual transformations associated with colour constancy, is unlikely to retrieve surface identity lost in this uncertain environment.

## Methods

### Irradiance fluctuations

An independent estimate of the minimum level of irradiance fluctuations was obtained from pyrheliometer recordings archived by the World Radiation Monitoring Center (WRMC). The station nearest to the site of the hyperspectral recordings used in this study was Cener in Sarriguren, Navarra, Spain^8,9^. Normal incidence recordings of surface irradiance were extracted for days in June and October containing fewest interruptions of the solar beam^36^. The standard deviation (SD) of the mean at 1-min intervals was derived by a method proposed by Rice for nonparametric residual variance estimates^65^. Its value divided by the mean, i.e. the relative SD, varied through the day. On 3 June 2010, it had a minimum of 0.13% at 13:00 and on 19 October 2010 a minimum of 0.09% at 13:00. On both days it remained less than 0.5% between 7:00 h and 16:00 but increased outside this interval.

### Hyperspectral radiance data

Eighteen pairs of unaveraged hyperspectral radiance images of outdoor vegetated and nonvegetated stationary scenes were extracted from sets of hyperspectral data collected from the Minho region of Portugal in 2002 and 2003. One consisted of 14 scenes from which single pairs of images were available separated by intervals of about 1 min to 15 min^12,19^. The other set consisted of four scenes from which multiple pairs of images were available separated by intervals of about 1 min up to 4.6 h^32^. Pairs of images were excluded if significant movement in the scene was detected during the acquisition or became obvious during subsequent image registration operations. Each image had dimensions 1344 × 1024 pixels and spectral range 400–720 nm sampled at 10-nm intervals. The angular subtense of each scene at the hyperspectral camera was approximately 6.9° × 5.3°, so that each pixel in the image represented the integrated image radiance over approximately 0.3 × 0.3 arcmin^32^.

The contribution of noise in the imaging system to differences in successive hyperspectral images was negligible in comparison with the effects of illumination change, even over 2 min, as illustrated by the plots in Fig. 1.

Colour renderings of one member of each image pair are shown in Fig. 2. Telespectroradiometer recordings of the correlated colour temperature of the direct illumination on a neutral reference surface in the field of view did not differ reliably between acquisitions up to 15 min apart. Reliable changes in correlated colour temperature, e.g. from 5949 K to 3014 K, were, however, recorded across much larger intervals of 4.6 h with the scene at the top right in Fig. 2.

Each hyperspectral image was registered over wavelength by uniform scaling and translation to compensate for variations in optical image size, especially at the ends of the spectrum. Each pair or sequence of hyperspectral images for each scene was then registered over acquisition time by translation to compensate for any residual differences in optical image position. All image registrations were performed to subpixel accuracy with in-house software. For some scenes, padding artefacts a few pixels wide were visible at the edges of the images, and were subsequently trimmed. Images were calibrated for spectral radiance against independent spectral radiance data recorded from a neutral reference surface or surfaces embedded in the scene or in the field of view^21^. Data from these image pairs has not been previously reported.

### Simulated illumination changes

As a control, a reflected radiance image $L\left( {u,v;\lambda } \right)$, indexed by spatial coordinates *u*, *v*, and wavelength *λ*, was represented as the product of an effective spectral reflectance $R\left( {u,v;\lambda } \right)$ and a global illuminant $E_{0} \left( \lambda \right)$, defined by a daylight with the same correlated colour temperature as the direct beam; that is, $L\left( {u,v;\lambda } \right) = E_{0} \left( \lambda \right)R\left( {u,v;\lambda } \right)\;.$ Notation follows previous use^21,38^. Given $R\left( {u,v;\lambda } \right)$ and two fixed daylight illuminants, $E_{1} \left( \lambda \right)$ and $E_{2} \left( \lambda \right)$, corresponding radiance images were then obtained as $L_{1} \left( {u,v;\lambda } \right) = E_{1} \left( \lambda \right)R\left( {u,v;\lambda } \right)\; = (E_{1} \left( \lambda \right)/E_{0} \left( \lambda \right))L\left( {u,v;\lambda } \right)$ and $L_{2} \left( {u,v;\lambda } \right) = E_{2} \left( \lambda \right)R\left( {u,v;\lambda } \right)\; = (E_{2} \left( \lambda \right)/E_{0} \left( \lambda \right))L\left( {u,v;\lambda } \right)$. The fixed daylight illuminants were drawn from around noon and towards the evening, with respective correlated colour temperatures of 6500 K and 4000 K^4,66^.

### Cone excitations

For demonstration only, the spectral radiance $L\left( {u,v;\lambda } \right)$ at time *t*, was converted to long-, medium-, and short-wavelength-sensitive cone excitations $q_{\text{L}} (u,v,t),\;q_{{\text{M}}} (u,v,t),\;q_{{\text{S}}} (u,v,t)$ in a standard way^38^. Integrals were evaluated numerically over the spectral range 400–720 nm in 10-nm steps. For each *k* = L, M, S, cone excitations $q_{k} (u,v,t)$ were subjected to von Kries scaling^7^ by the corresponding spatial mean $\overline{q}_{k} (t)$ of the sample points; that is, $q^{\prime}_{k} (u,v,t) = {{q_{k} (u,v,t)} \mathord{\left/ {\vphantom {{q_{k} (u,v,t)} {\overline{q}_{k} (t)}}} \right. \kern-\nulldelimiterspace} {\overline{q}_{k} (t)}}$. Sample areas were defined by a thin horizontal strip, size 896 × 30 pixels. At each point $(u,v)$ in the sample, the magnitude of the physical difference in cone excitations at times *t*_1_ and *t*_2_, i.e. $\Delta q^{\prime}_{k} (u,v) = q^{\prime}_{k} (u,v,t_{2} ) - q^{\prime}_{k} (u,v,t_{1} )$, was summarized by the unweighted Euclidean norm $\Delta e(u,v) = \left[ {\sum\nolimits_{k} {\Delta q^{\prime}_{{\text{k}}} (u,v)^{2} } } \right]^{1/2}$. The SD of $\Delta e(u,v)$ was then evaluated over $(u,v)$ and the result recorded as a function of the vertical position of the strip in the 1344 × 1024-pixel image. Because of von Kries scaling, the SD was defined relative to the mean of the sample.

### Uniform colour spaces

Hyperspectral radiance images were represented in the approximately uniform colour space CIECAM02^38^ and, for comparison, S-CIELAB^41^, where S-CIELAB is an extension of CIELAB that incorporates pattern-separable spatial filtering (https://github.com/wandell/SCIELAB-1996/). The spectral radiance $L\left( {u,v;\lambda } \right)$ was converted to normalized tristimulus values and then into CIECAM02 coordinates and into CIELAB coordinates for S-CIELAB space^39,40^. The coordinates of CIECAM02 are $J,a_{{\text{C}}} ,b_{{\text{C}}}$, where $J$ correlates with lightness, ranging from 0 to 100, and $a_{{\text{C}}}$ and $b_{{\text{C}}}$ correlate with redness–greenness and yellowness–blueness, respectively. The corresponding coordinates of CIELAB are $L^{*} ,a^{*} ,b^{*}$. There are, however, differences in the degree of uniformity of CIECAM02 and CIELAB spaces^67^. Their physiological plausibility has been evaluated with electroencephalographic and magnetoencephalographic methods^68^.

### Observer uncertainty

The effect of uncertainty in the observer was modelled in CIECAM02 and S-CIELAB spaces as internal additive noise^20,69^. The underlying probability density function or pdf was assumed to be either Gaussian or uniform, with the latter providing a link to the deterministic or hard discrimination thresholds Δ*E*^thr^ used in colorimetry^7^. A just perceptible colour difference corresponds to a value for Δ*E*^thr^ of 0.5 in CIECAM02 space, which is approximately equivalent^44^ to a value of 1.0 in CIELAB space^40,70,71^, though larger values may be defined by acceptability criteria^62^ and in categorization tasks^16^. Memory effects are not considered here^55,56^.

The Gaussian and uniform distributions were each parameterized by a nominal width *w*, which was referred^47^ to values of Δ*E*^thr^. For a Gaussian distribution with SD $\sigma$, say, $w$ was defined as $12^{1/2} \sigma$. For a uniform distribution, $w$ was defined as the width of the support of the distribution. Since the SD of the uniform distribution is $12^{ - 1/2} w$, Gaussian and uniform distributions with the same $w$ then have the same variance. Values of Δ*E*^thr^ were set to 0.5 and 1.0 in CIECAM02 space and to 1.0 in S-CIELAB space.

### Mutual information and distinguishable points

The method used to estimate the number of surfaces distinguishable by their colour follows previous studies^20,21,47^. Scenes were not segmented into regions, except for the trivial limit defined by pixel resolution. Critically, points are assumed to be drawn randomly from each scene^29^. At each instant, the triplets $J,a_{{\text{C}}} ,b_{{\text{C}}}$ in CIECAM02 space or $L^{*} ,a^{*} ,b^{*}$ in S-CIELAB space can be treated as instances ${\mathbf{a}}$, say, of a three-dimensional continuous random variable ${\mathbf{A}}$ with pdf $f({\mathbf{a}})$, which underlies the observed distribution of colours in the scene. Notice that the spatial filtering associated with S-CIELAB is applied before the random draw rather than afterwards.

The uncertainty in the random variable ${\mathbf{A}}$ is quantified^28^ by the Shannon differential entropy $h({\mathbf{A}})$ given by

1 $$h({\mathbf{A}}) = - \int {f({\mathbf{a}})\log f({\mathbf{a}})} \;{\text{d}}{\mathbf{a}},$$

which is measured in bits if the logarithm is to the base 2 (the symbols $h$ and ${\mathbf{a}}$ should not be confused with colorimetric quantities). When *f* is the uniform function, that is, when the colours are equally probable, the entropy coincides with the logarithm of the conventional volume of the colour gamut.

Observer responses can also be treated as instances of a three-dimensional continuous random variable, ${\mathbf{B}}$ say. The amount of information that ${\mathbf{B}}$ provides about ${\mathbf{A}}$ is given by the mutual information^28^, written $I({\mathbf{A}};{\mathbf{B}})$. Its inverse logarithm may be interpreted as the approximate number of points *N* in the scene that can be distinguished in the presence observer uncertainty; that is,

$$N = 2^{{I({\mathbf{A}};{\mathbf{B}})}}.$$

To evaluate $I({\mathbf{A}};{\mathbf{B}})$, it can be expressed as a combination of the differential entropies $h({\mathbf{A}})$, $h({\mathbf{B}})$, and $h({\mathbf{A}},{\mathbf{B}})$, where $h({\mathbf{A}},{\mathbf{B}})$ is the differential entropy of ${\mathbf{A}}$ and ${\mathbf{B}}$ taken jointly; that is,

2 $$I({\mathbf{A}};{\mathbf{B}}) = h({\mathbf{A}}) + h({\mathbf{B}}) - h({\mathbf{A}},{\mathbf{B}}).$$

Suppose that observer internal noise is represented by a three-dimensional continuous random variable ${\mathbf{W}}$, so that ${\mathbf{B}} = {\mathbf{A}} + {\mathbf{W}}$. If ${\mathbf{W}}$ is obtained by drawing pseudorandom values from the assumed noise distribution, the right hand side of (2) can be estimated numerically. But using histograms in place of the unknown pdf *f* in (1) can lead to biases in estimates. Instead, the more accurate Kozachenko-Leonenko *k*th-nearest-neighbour estimator^72,73^ was used in conjunction with an offset method^29^ (https://github.com/imarinfr/klo). Though this calculation sets a limit on the number of distinguishable points, the number of distinguishable surfaces cannot exceed this limit^43^.

### Mutual information and identifiable points

The method used to estimate the number of surfaces identifiable by their colour after an interval is similar. At times $t_{1}$ and $t_{2}$, the triplets in CIECAM02 space or S-CIELAB space are treated as instances of three-dimensional continuous random variables ${\mathbf{A}}_{1}$ and ${\mathbf{A}}_{2}$. If observer responses are treated as instances of a three-dimensional continuous random variable ${\mathbf{B}} = {\mathbf{A}}_{2} + {\mathbf{W}}$, then the amount of information that ${\mathbf{B}}$ provides about ${\mathbf{A}}_{1}$ is given by the mutual information $I\left( {{\mathbf{A}}_{1} ;{\mathbf{B}}} \right) = I\left( {{\mathbf{A}}_{1} ;{\mathbf{A}}_{2} + {\mathbf{W}}} \right)$, which can be estimated as before. Its inverse logarithm may be interpreted as the approximate number of points *N* in the scene that can be identified between times $t_{1}$ and $t_{2}$ in the presence observer uncertainty.

For the special case in which the time interval is zero, so that $t_{1} = t_{2}$ and ${\mathbf{A}}_{1} = {\mathbf{A}}_{2} = {\mathbf{A}}$, say, the inverse logarithm of the mutual information $I\left( {{\mathbf{A}};{\mathbf{B}}} \right) = I\left( {{\mathbf{A}};{\mathbf{A}} + {\mathbf{W}}} \right)$ reduces to the number of distinguishable points. An intuitive rationale for this interpretation is provided elsewhere^20,21^. This relationship between distinguishability and identification across time does not imply that the same processes necessarily mediate observer judgements^74^.

Although it might seem counterintuitive, *N* is not presented as a proportion of the total number of points in the scene, defined in some way, since *N* is independent of sample size providing that the sample is sufficiently large. This independence can be confirmed empirically^18^ by plotting the variation in the number of points identified by nearest-neighbour matching^29^ as sample size is progressively increased: the number of matched points asymptotes below what is theoretically possible.

Because of the linearizing effect of a logarithmic scale on estimates of *N*, means and 95% BCa confidence limits^75^ over scenes were calculated for the corresponding values of mutual information and then inverse logarithms taken.

## Supplementary Information

Supplementary Tables.

## Data Availability

Hyperspectral radiance data analysed in this study are available at https://doi.org/10.6084/m9.figshare.c.5240420 and at https://personalpages.manchester.ac.uk/staff/d.h.foster/ and https://sites.google.com/view/sergionascimento/home/scientific-data. Software for estimating differential entropy and mutual information for multivariate data is available at https://github.com/imarinfr/klo.
