# Supplementary material for: Fluctuating environmental light limits number of surfaces visually recognizable by colour
Source: Sci Rep. 2021 Jan 22;11:2102. doi: 10.1038/s41598-020-80591-9 (PMC7822868; doi:10.1038/s41598-020-80591-9)
Supplement: Supplementary file 1 — Supplementary Tables. [file 41598_2020_80591_MOESM1_ESM.docx]

**Fluctuating environmental light limits number of surfaces visually recognizable by colour**

David H. Foster

Department of Electrical and Electronic Engineering

University of Manchester
Manchester M13 9PL, UK

Email d.h.foster@manchester.ac.uk

## SUPPLEMENTARY INFORMATION

Fluctuating environmental light limits number of surfaces visually recognizable by colour. Au: David H. Foster

### Supplementary Table S1. Numbers of distinguishable surfaces and surfaces identifiable over time intervals (CIECAM02 colour space, reference threshold Δ*E*^thr^ = 0.5) ^a^

|  |  |  |  | **Observer internal noise^b^** | |
| --- | --- | --- | --- | --- | --- |
|  | **Surface measure** | **Images/scene^c^** | **Interval** | **Gaussian** | **Uniform** |
| 18 scenes^d^ | Distinguishability | 1 | – | 12000 (8200, 17000) | 19000 (13000, 27000) |
|  | Identifiability over intervals | 2 | 1–15 min | 270 (180, 400) | 270 (180, 410) |
| 4 scenes^e^ | Distinguishability | >17 | – | 9500 (7400, 11000) | 15000 (12000, 18000) |
|  | Identifiability over intervals | >110 | 2 min | 580 (480, 700) | 580 (480, 700) |
|  |  |  | 10 min | 210 (120, 310) | 210 (120, 310) |
|  |  |  | 1 h | 69 (26, 120) | 69 (26, 120) |

^a^ Entries are logarithmic inverses of mutual information estimates averaged over images, image pairs, and regression estimates. Estimated 95% BCa confidence limits in parentheses were based on 5000 bootstrap samples^75^. All entries to 2 significant figures.

^b^ Gaussian and uniform models of observer internal noise were referred to a hard discrimination threshold Δ*E*^thr^.

^c^ Number of images from each scene acquired at different times.

^d^ All scenes in Fig. 2.

^e^ Top row scenes in Fig. 2.

### Supplementary Table S2. Numbers of distinguishable surfaces and surfaces identifiable over time intervals (S-CIELAB colour space, reference threshold Δ*E*^thr^ = 1.0) ^a^

|  |  |  |  | **Observer internal noise^b^** | |
| --- | --- | --- | --- | --- | --- |
|  | **Surface measure** | **Images/scene^c^** | **Interval** | **Gaussian** | **Uniform** |
| 18 scenes^d^ | Distinguishability | 1 | – | 760 (500, 1400) | 1200 (840, 2200) |
|  | Identifiability over intervals | 2 | 1–15 min | 560 (390, 820) | 660 (460, 950) |
| 4 scenes^e^ | Distinguishability | >17 | – | 490 (330, 850) | 810 (570, 1400) |
|  | Identifiability over intervals | >110 | 2 min | 390 (270, 630) | 480 (350, 750) |
|  |  |  | 10 min | 230 (180, 290) | 270 (220, 340) |
|  |  |  | 1 h | 130 (120, 170) | 150 (120, 190) |

^a^ Details as for Supplementary Table S1.

### Supplementary Table S3. Numbers of distinguishable surfaces and surfaces identifiable over time intervals (CIECAM02 colour space, reference threshold Δ*E*^thr^ = 1.0) ^a^

|  |  |  |  | **Observer internal noise^b^** | |
| --- | --- | --- | --- | --- | --- |
|  | **Surface measure** | **Images/scene^c^** | **Interval** | **Gaussian** | **Uniform** |
| 18 scenes^d^ | Distinguishability | 1 | – | 1600 (1200, 2400) | 2600 (1900, 3800) |
|  | Identifiability over intervals | 2 | 1–15 min | 180 (120, 240) | 180 (120, 240) |
| 4 scenes^e^ | Distinguishability | >17 | – | 1400 (1100, 1600) | 2200 (1800, 2600) |
|  | Identifiability over intervals | >110 | 2 min | 310 (250, 350) | 310 (260, 370) |
|  |  |  | 10 min | 130 (81, 170) | 130 (81, 170) |
|  |  |  | 1 h | 47 (16, 72) | 47 (20, 80) |

^a^ Details as for Supplementary Table S1.
